# ERG Transcriptional Networks in Primary Acute Leukemia Cells Implicate a Role for ERG in Deregulated Kinase Signaling

**DOI:** 10.1371/journal.pone.0052872

**Published:** 2013-01-03

**Authors:** Juliane Bock, Liliana H. Mochmann, Cornelia Schlee, Nasrin Farhadi-Sartangi, Stefanie Göllner, Carsten Müller-Tidow, Claudia D. Baldus

**Affiliations:** 1 Department of Hematology and Oncology, Charité, University Hospital Berlin, Campus Benjamin Franklin, Berlin, Germany; 2 Department of Medicine, Hematology, Oncology and Pneumology, University of Münster, Münster, Germany; University of Nebraska Medical Center, United States of America

## Abstract

High expression of the E26 transforming sequence related gene (ERG) is associated with poor prognosis in a subgroup of leukemia patients with acute myeloid (AML) and acute T-lymphoblastic leukemia (T-ALL). In a previous study we proposed that *ERG* overexpression may deregulate several signaling cascades in acute leukemia. Herein, we further expand those studies by identifying a consensus of biological targets in primary blasts of newly diagnosed acute leukemia patients. Our findings of chromatin immunoprecipitation-on-chip of primary samples revealed 48 significantly enriched single genes including *DAAM1* and *NUMB*. Significantly enriched signaling pathways included WNT/β-catenin, p53, and PI3K/AKT with *ERG* overexpression inducing dephosphorylation of AKT(Ser473) relative to non *ERG* expressing K562 cells. Cell based *ERG* overexpression studies also revealed drug resistance to multi-kinase inhibitor, BAY 43-9006 (Sorafenib) and to the tyrosine kinase inhibitor TKI258. Thus in primary leukemic cells, ERG may contribute to the dysregulation of kinase signaling, which results in resistance to kinase inhibitors.

## Introduction

The erythroblastosis virus E26 transforming sequence family member, ERG, plays an important role in early hematopoiesis and hematopoietic stem cell (HSC) maintenance [1], [2]. ERG is also part of fusion proteins in solid tumors such as prostate cancer [3], [4], Ewing Sarcoma [5] and in acute myeloid leukemia (AML) [6]. In acute leukemia, high *ERG* mRNA expression levels are an independent prognostic factor. Studies have demonstrated that increased *ERG* expression is associated with poor prognosis in cytogenetically normal AML [7], [8], [9] and in adult T-ALL [10]. Collectively, the data show that ERG is required for normal hematopoiesis, but when deregulated promotes leukemogenesis.

ERG dependent deteriorating effects are mirrored in *in vivo* mouse models, where ectopic ERG expression in hematopoietic progenitors resulted in megakaryoblastic leukemia [11]. Further studies demonstrated that elevated *ERG* expression, in cooperation with *NOTCH1* mutations, promotes T-ALL in mice [12]. Also, *ERG* overexpression in mice induced expansion of erythroblasts, T-cells and B-cell precursors, while inhibiting differentiation of B-cells [13].

ERG regulated pathways and biological functions are largely unmapped; thus further investigations are necessary to improve our understanding of ERG mediated leukemogenesis. Recently, we reported a genome-wide screen of ERG specific target genes in acute T-lymphoblastic Jurkat cells and identified the non-canonical WNT gene, *WNT11*, as a direct target of ERG [14]. To further verify and expand the present knowledge on ERG regulated molecular functions, we now performed chromatin immunoprecipitation-on-chip (ChIP-chip) analyses in primary acute leukemia samples (AML and T-ALL). Due to enrichment of the PI3K/AKT signaling cascade, we further explored the sensitivity to kinase inhibitors inhibitors BAY 43-9006 (Sorafenib) and tyrosine kinase inhibitor TKI258 (Dovotinib). Our results demonstrate that *ERG* overexpression induces resistance to kinase inhibitors including Sorafenib and TKI258.

## Materials and Methods

### Patient Samples and Cell Culture

Bone marrow samples from newly diagnosed acute leukemia patients (5 AML and 1 T-ALL) and one bone marrow sample from a healthy donor (nBM) were used for ChIP assays. Clinical and molecular characteristics are shown in Table S1. Written consent was obtained from all donors according to the Declaration of Helsinki and approved by the Berlin Ethics Committee. Fresh bone marrow aspirates were enriched for the mononuclear fraction by Ficoll-Hypaque density gradient (Amersham Pharmacia Biotech, Uppsala, Sweden). Additionally, ChIP-chip assays using HL60 cells, a promyelocytic cell line, which does not express *ERG*, were conducted to serve as a negative control. The human chronic myeloid leukemia cell line K562, the T-ALL cell line Jurkat and the cell line HL60 were obtained from the German Resource Center for Biological Material, DSMZ (Braunschweig, Germany) and grown in RPMI media with 10% fetal bovine serum at 37°C in a 5% CO_2_ humidified chamber.

### Chromatin Immunoprecipitation

ChIP experiments of all samples were performed as described [15]. Briefly, 5×10^8^ cells were crosslinked with formaldehyde at a final concentration of 1%. Subsequently, cells were lysed with RIPA buffer and DNA was fragmented by sonication to a fragment size range of 200 to 1000 bp with a mean of 500 bp. The fragmented DNA lysate was precleared before the addition of 3 µg of antibodies per 0.5 mg of protein lysate. The following antibodies were used in duplicate experiments for each sample: Erg-1/2/3 C20 (epitope at C-terminus) and a non-specific IgG antibody as control (Santa Cruz Biotechnologies, Santa Cruz, CA). For each group, reverse crosslinking and DNA purification were performed. Two input controls were carried through for each sample.

### DNA Amplification and Fluorescence Labeling

DNA amplification was carried out in two steps: first, using the commercially available GenomePlex Whole Genome Amplification (WGA) Kit (Sigma-Aldrich, St Louis, MO) according to the manufacturer’s instructions. Second, enriched ChIP DNA was labeled using the amino-allyl-conjugated dUTP labeling kit BioPrime (Invitrogen, Carlsbad, CA) and a random primer. Subsequently, the products were purified and conjugated with monofunctional NHS-ester Alexa Fluor 647 Dye (red) for input control and Alexa Fluor 555 Dye (yellow-green; Invitrogen, Carlsbad, CA) for ERG or IgG.

### Microarray Hybridization

The promoter array used contains more than 11,000 human promoters represented by approximately 35,000 oligonucleotides as described [16]. Input control and experimental group (ERG or IgG respectively) were differentially labeled and co-hybridized to a promoter array as described [17]. Four hybridizations were carried out for each primary sample and HL60 control (Table 1).

**Table 1:** Scheme of ChIP-chip hybridization per sample.

Hybridization #	Hybridized Templates	Target genes
Hybridization 1	input-1 (Alexa 647)/IgG-1(Alexa 555)	unspecific genes
Hybridization 2	input-2 (Alexa 647)/IgG-2(Alexa 555)	
Hybridization 3	input-1 (Alexa 647)/ERG-1(Alexa 555)	ERG enriched genes
Hybridization 4	input-2 (Alexa 647)/ERG-2(Alexa 555)	

For each primary sample four hybridizations were carried out. IgG-enriched DNA was paired with input-DNA and ERG-enriched with input-DNA. DNA-mixtures were hybridized to a promoter chip array. Thus, genes identified as enriched in the IgG/input hybridizations (hybridization 1 and 2) were treated as unspecifically enriched genes and those identified in the ERG/input hybridizations (hybridization 3 and 4) were regarded as ERG-enriched genes. Finally, only those genes that were enriched in hybridization 3 and 4 only were regarded as putative ERG target genes.

### Statistical Analysis

Microarrays were scanned using the Spotreader software (Niles Scientific, Portola Valley, CA). Spots with intensities below threshold or abnormal signals were flagged. The data were normalized by the median over the entire array for the log_2_(ChIP/Input). Using BRB Array Tools software (National Cancer Institute, http://linus.nci.nih.gov/BRB-ArrayTools.html), the results for each spot (two log_2_ ratios for ERG over Input and two log_2_ ratios for IgG over Input) were analyzed by a class comparison identifying differentially enriched genes between ERG and IgG experimental group.

### Bioinformatic Analyses

Data sets were analyzed with Ingenuity Pathways Analysis (Ingenuity Systems, www.ingenuity.com) and DAVID Bioinformatic Resources 6.7, National Institute of Allergy and Infectious Diseases (NIAID), NIH (http://david.abcc.ncifcrf.gov/) [18]. Molecules from the dataset with a P-value <0.05 and an enrichment ratio >1.25 were considered for the analysis. Fisher’s exact P-value and Benjamini-Hochberg multiple correction testing was used. Analysis of transcription factor binding site motifs was performed using Pscan [19].

### Quantitative Real-time PCR Analyses

For the validation of promoter ChIP enrichment, genes chosen for validation by PCR included *NUMB* and *DAAM1* which are 5′- AGAGATGAGGAGCAGCAGGT-3′ paired with 5′- CCCATCCCAGCTCAGCTAT-3′, and 5′-CCGCTAATAGATTGGGACCAC-3′ paired with 5′-CAATGGGATCCGGTTAAACA-3′, respectively. SYBR Green (Invitrogen GmbH, Karlsruhe, Germany) based PCR was applied to test for the relative amount of ChIP enriched DNA fragments over enrichment by unspecific IgG. The cut off for a true enrichment was set at 1.5-fold over IgG in at least one experimental group.

### *ERG* Overexpression Studies

To test for ERG dependent proliferative effects, K562 cells were used carrying a two vector system consisting of pTet-on Advanced (pTet), pTRE-Tight-BI-DsRedExpress (pTRE), expressing *ERG* and DsRed fluorescence, as previously described [15]. *ERG* induction, upon treatment with doxycycline (dox) 1 µg/ml was verified by red fluorescence detection (DsRed), reverse transcriptase real-time PCR and western blotting. In addition, Jurkat cells were transiently transfected with AMAXA (Lonza Cologne AG, Cologne, Germany) according to manufacturer’s instructions. The transient transfections were carried out using the construct pDom-empty or pDom-*ERG*, both derivatives of pcDNA3. The construct pDom-*ERG* contains the *ERG* coding region between the pcDNA3 EcorI-XhoI restriction sites.

### Cell Proliferation

In order to measure cell proliferation, WST-1 reagent was used according to the manufacturer’s instructions (Roche Diagnostics GmbH, Mannheim, Germany). Transfected pDom-*ERG* and pDom-empty control cells were seeded into a 96-well plate. Tet-transfected K562 cells were cultured and *ERG* expression was induced by the addition of dox for a period of 72 hours prior to seeding in a 96-well plate. *ERG* overexpressing and control cells were seeded at a concentration of 3×10^4^ cells/well. After 24 hours cells were treated with the multi-kinase inhibitor BAY 43-9006 (Sorafenib), purchased from Enzo Life Sciences (Farmingdale, NY), at a concentration of 10 µM, or with the tyrosine kinase inhibitor TKI258 (Dovitinib, kind gift from Novartis Pharma AG, Basel, Switzerland) at a concentration of 1 µM. The color conversion for the measurement of proliferation was measured 48 and 72 hours after the addition of TKI258 and Sorafenib. Absorbance was measured at 450 nm following a 60 minute incubation with WST-1 reagent.

### Flow Cytometry

Annexin V-FITC apoptosis assay (BD Biosciences, San Diego, CA) was used according to manufacturer’s instructions. Intracellular stain of pan AKT and phosphorylated AKT(Ser473) in K562 and Jurkat cells were performed by a 10 minute fixation, 15 minute methanol permeabilization, and staining at 1∶100 for both anti-pan AKT and anti-AKT(Ser473) Alexa 647. Secondary antibody, anti-mouse Alexa488 IgG was used for detection of pan AKT. The three antibodies were purchased from Cell Signaling (Davers, MA). Flow cytometry measurements were performed with Becton Dickinson FACSCalibur.

## Results

### Identification of ERG Target Genes in Acute Leukemia

**Figure 1:**
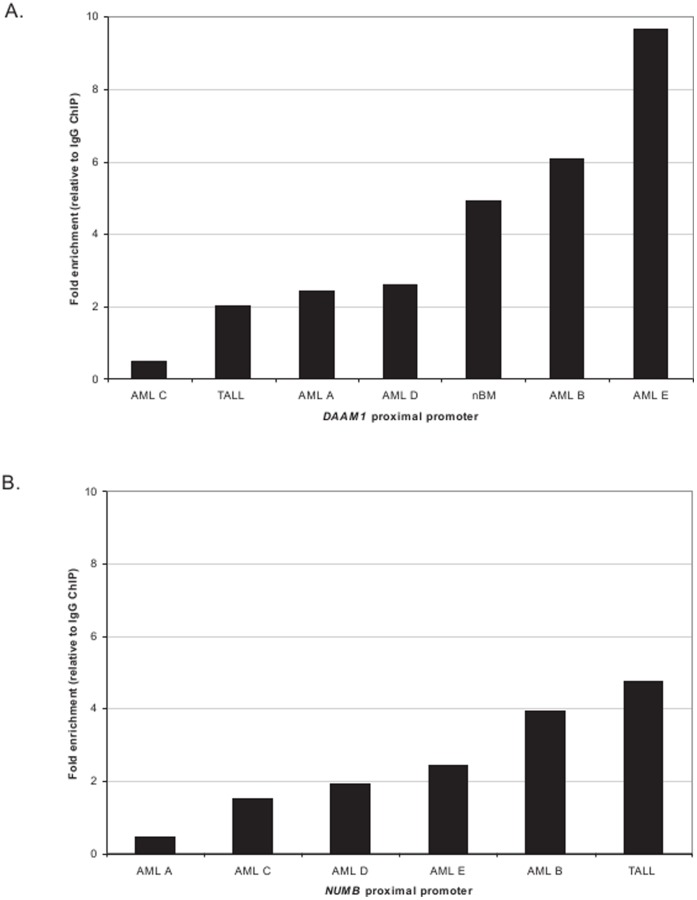
Promoter ChIP enrichment of *DAAM1* and *NUMB* verified by quantitative real-time PCR analysis. **A and B).** Quantitative PCRs with primers designed to include the conserved ETS binding sequence in the proximal promoter of *DAAM1* and *NUMB* were applied. The *DAAM1* and *NUMB* promoters were enriched 1.5-fold over IgG control in six ChIPs and in five ChIPs, respectively (one of two representative experiments is shown).

Previously, we conducted a genome wide screen of ERG transcriptional targets that revealed a significant cluster of genes belonging to the WNT signaling pathway. Herein, we have conducted ChIP-chip assays on fresh primary AML samples (n = 5, AML A-E), one primary T-ALL (T-ALL) and one normal bone marrow sample (nBM) to broaden our scope of potentially novel targets and deregulated pathways. To validate ChIPs, we performed quantitative real-time PCR of the WNT gene, *Disheveled-associated activator of morphogenesis 1* (*DAAM1*), co-expressed with *ERG* in prostate cancer [20]. *DAAM1* was enriched in 6 ChIPs with an at least two-fold enrichment over IgG control indicating that *DAAM1* is an additional putative ERG target in acute leukemia (Figure 1A).

**Figure 2:**
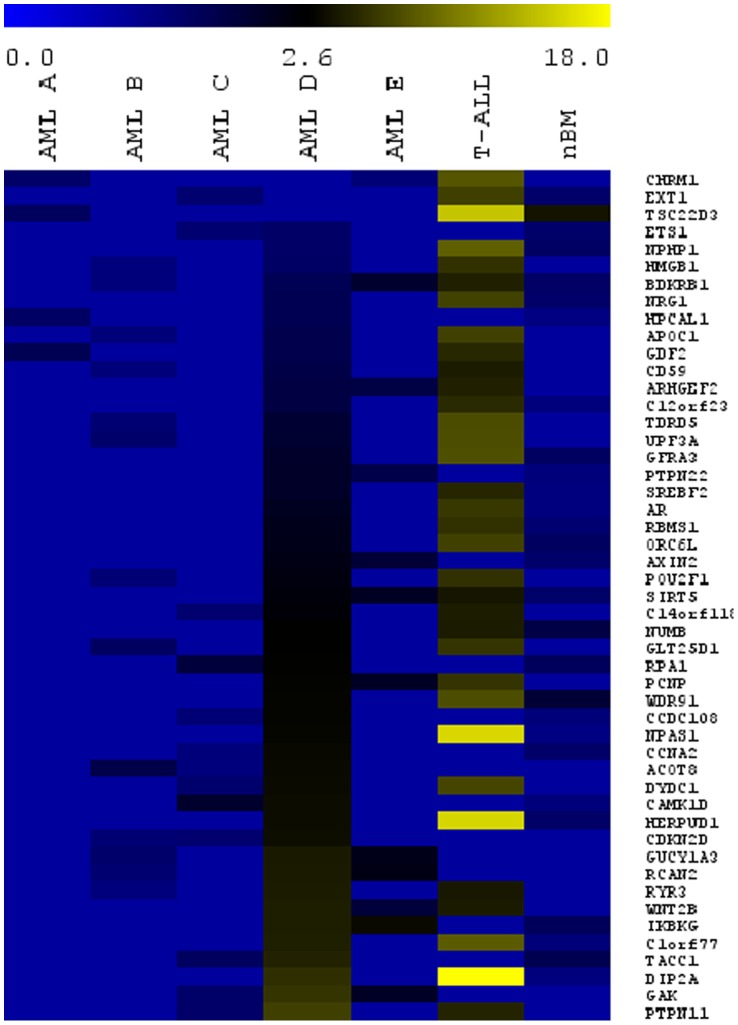
The significant genes derived from 7 ChIP-chip microarray data are displayed with the use of MultiExperiment Viewer graphical interface. The fold enrichment for 48 genes shared by at least 3 of ChIP-chips was uploaded to MultiExperiment Viewer to obtain a heat map representing the gene overlap of all ChIP-chips. Significantly enriched genes are >1.25 fold change with respect to the IgG control. The overlap of 48 unique genes shows that AML D, T-ALL and nBM share a greater number of enriched target genes. AML A-E are ordered with respect to *ERG* mRNA expression and genes are ranked according AML D P-Values.

For a broader screen, ChIP DNA obtained from AML A-E, T-ALL, nBM and the HL60 negative control were individually hybridized to a promoter chip array. Significantly enriched single genes as determined by the BRB Array Tools (fold enrichment >1.25, p<0.05) from 7 ChIP-chips included: 55 from AML A, 253 from AML B, 256 from AML C, 3113 from AML D, 167 from AML E, 951 from T-ALL, and 510 from nBM (Table 2). The negative control HL60 (lacking detectable levels of *ERG* mRNA) yielded 253 genes, thus in the subsequent analyses these unspecific genes were subtracted as false positive hits. A single consensus gene set of the 7 ChIP-chips was not obtainable despite lowering fold enrichment to 1.25, which was likely due to the heterogeneous molecular background of the leukemic samples (see Table S1) as well as the experimental variability of the ChIP-chip multi-step process. We did, however, find a considerable overlap of genes shared between ChIP-chips performed with AML D and T-ALL (257 genes), T-ALL and nBM (49 genes) and AML D and nBM (152 genes). Moreover, ChIP-chip assays yielded a total of 48 genes enriched in at least three of the seven primary samples including genes associated with cell cycle such as *ARGHEF2*, *CCNA2*, *CDKN2D*, *GAK2*, *ORCL6*, *PCPNP*, and *TACC1* and genes associated with cancer such as *AR*, *AXIN2*, *IKBKG*, *ETS1*, *PTPN11,* and *WNT2B* (Figure 2).

**Table 2:** ChIP-chip enriched genes.

	AML A	AML B	AML C	AML D	AML E	T-ALL	nBM	HL60
*ERG* mRNA expression	0.17	0.26	0.68	1.02	1.23	0.38	0.18	0
Gene count	55	253	256	3113	167	951	510	253

Displayed are 8 ChIP-chip samples with the *ERG* mRNA expression measured by quantitative real-time PCR as well as the number of significantly enriched single genes, identified by ChIP-chip using BRB Array Tools (fold enrichment >1.25, p<0.05), for primary AML samples (AML A through AML E), for T-ALL (T-ALL), a normal bone marrow sample (nBM) and the cell line HL60.

To identify transcription factor binding site motifs enriched among the gene lists identified by ChIP-chip, we applied the Pscan software for motif finding [19]. Enriched gene sets were uploaded and the region of −950 to +50 bp surrounding transcription start site (TSS) of each gene was used. In this region 6 out of the 7 primary samples showed enrichment for the ETS binding sequence 5′-GGAA/T-3′ (Table 3). AML B and the HL60 negative control showed no enrichment of transcription factor binding sites. The lack of motif enrichment among the target gene list of AML B might be due to a high degree of false positive enrichment, which could mask enrichment for the ETS motif. Moreover, additional transcription factor binding motifs were represented in AML D, T-ALL and the nBM. In the dataset generated for AML D, the binding motif REL (p-value = 0.0001) and the bZIP (p-value = 0.004) were overrepresented. In the dataset generated for T-ALL, the bHLH motif (p-value = 0.04) was overrepresented and for nBM dataset, the bZIP family motif (p-value = 0.02). These might account for co-regulation of additional genes with ERG at the promoter level. The results of the ETS motif enrichment for AML D are displayed graphically as an example (Figure S1A). The P-value of 9.5×10^−15^ (determined by z-test) confirms the high significance of ETS as overrepresented motif. These results validated that ChIP-chips using an ERG specific antibody in primary AML and T-ALL blasts resulted in the enrichment of ERG target genes harboring the ETS consensus sequence.

**Table 3:** Motif analysis of enriched gene lists.

ChIP	Significantly enriched motifs	P-Value
AML A	ETS	0.05
AML B	None	
AML C	ETS	0.03
AML D	ETS	9.50E-15
	REL	0.0001
	bZIP	0.004
AML E	ETS	0.02
T-ALL	ETS	0.04
	bHLH	0.04
nBM	ETS	0.02
	bZIP	0.001
HL60	None	

The table represents significantly enriched transcription factor binding motifs, with the P-Value and the Bonferroni P-Value. Gene lists from ChIP-chip analyses were analyzed for enrichment of transcription factor binding sites (motifs) using Pscan [22]. The analysis of promoters of enriched genes showed an enrichment of the ETS binding motif GGAA/T in the primary samples for the region −950 to +50 bp with regard to the transcription start site (TSS). AML B and the HL60 negative control were not enriched for ETS binding motifs. Furthermore, AML D and T-ALL showed an additional enrichment for further TF binding motifs.

### Molecular Functions and Pathways Regulated by ERG

**Figure 3:**
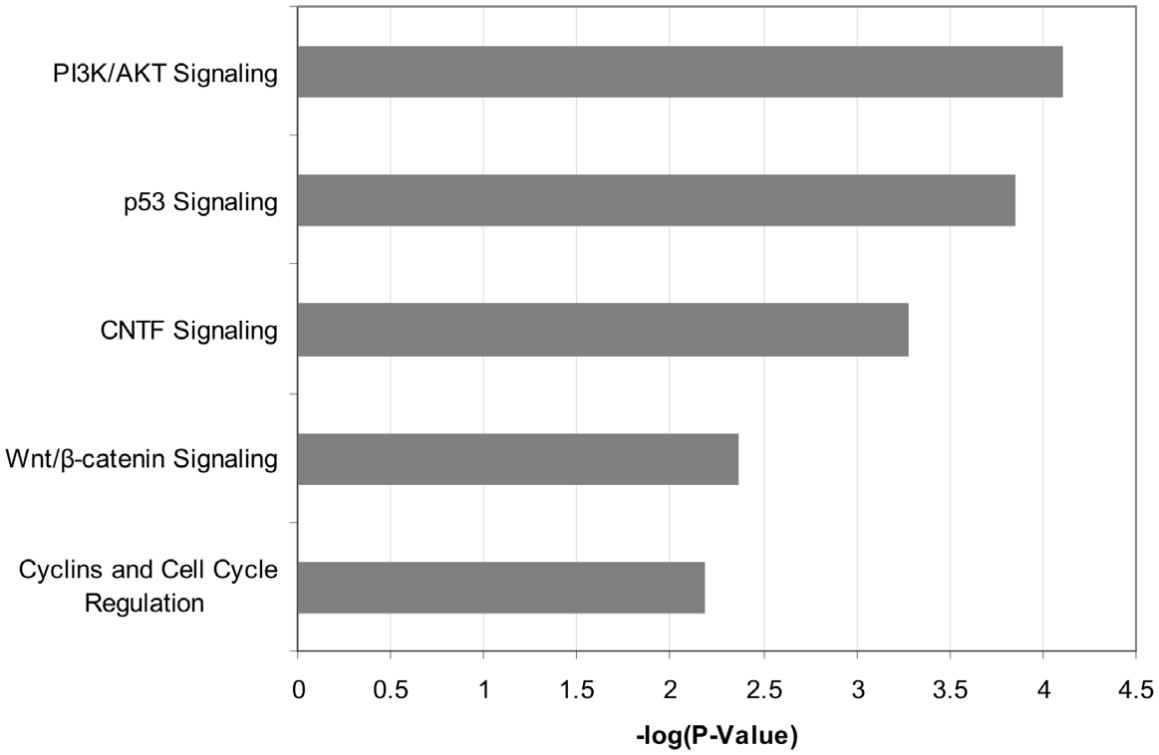
Biological pathways enriched in primary leukemia ChIP-chip analyses. Displayed are the biological pathways significantly enriched in at least three of seven primary leukemia samples by ChIP-chip. The bars represent the negative logarithm function of Fisher’s P-value (P<0.05).

To further identify molecular functions and pathways regulated by ERG, the ERG enriched target genes were analyzed with Ingenuity Pathways Analysis. Significant results with a P-value <0.05 and enrichment in at least 3 of the 7 ChIP-chip samples were considered. Unspecific functions and pathways enriched in ChIP-chip of the ERG negative cell line HL60 were removed to ensure specificity. The majority of enriched biological themes were associated with cellular processes such as growth and proliferation, cellular development, cell morphology, cellular movement, gene expression, and small molecule biochemistry (Figure S1 B). Interestingly, in nBM only molecules associated with cell cycle (B-H p-value = 0.008) and tissue development (B-H P-value = 0.02) were enriched, thus consistent with physiological functions regulated by ERG in non-malignant cells [1], [21]. In accordance to the enriched global functions, canonical pathways that were enriched in at least 3 samples (with a P-value <0.05) included PI3K/AKT, p53 signaling, and WNT signaling pathways (Figure 3). Interestingly, six of 48 genes listed in Figure 2 (*AR, DIP2A, GRFA3, NUMB, NRG1,* and *SREBF2*) are closely associated with PI3K/AKT signaling [22], [23], [24], [25], [26], [27]. For instance, NUMB antagonizes the NOTCH1 mediated activation of AKT [25] and the expression of *AR* is decreased by activation of AKT [28]. Also, AKT synergizes with SREBP, and assumingly with SREBF2, in activating gene transcription [27]. Quantitative promoter PCR was performed in selected, potential ERG targets genes related to PI3K or leukemia including *AR*, *NUMB*, *NRG1*, and *ETS1* in AML A-E, and T-ALL ChIPs. We uncovered that only the proximal promoter of *NUMB* was significantly enriched at least 1.5-fold in 5 of 6 leukemia ChIPs (Figure 1B).

Importantly, significantly overrepresented protein domains identified using DAVID Bioinformatic Resources 6.7 were protein kinases (p-value = 2.6×10^−5^) and serine/threonine kinases (p-value = 0.001). Thus, these data collectively reveal that ERG may influence the transcriptional regulation of kinase cascades such as PI3K/AKT signaling.

### ERG Overexpression Results in Dephosphorylation of AKT(Ser473)

**Figure 4:**
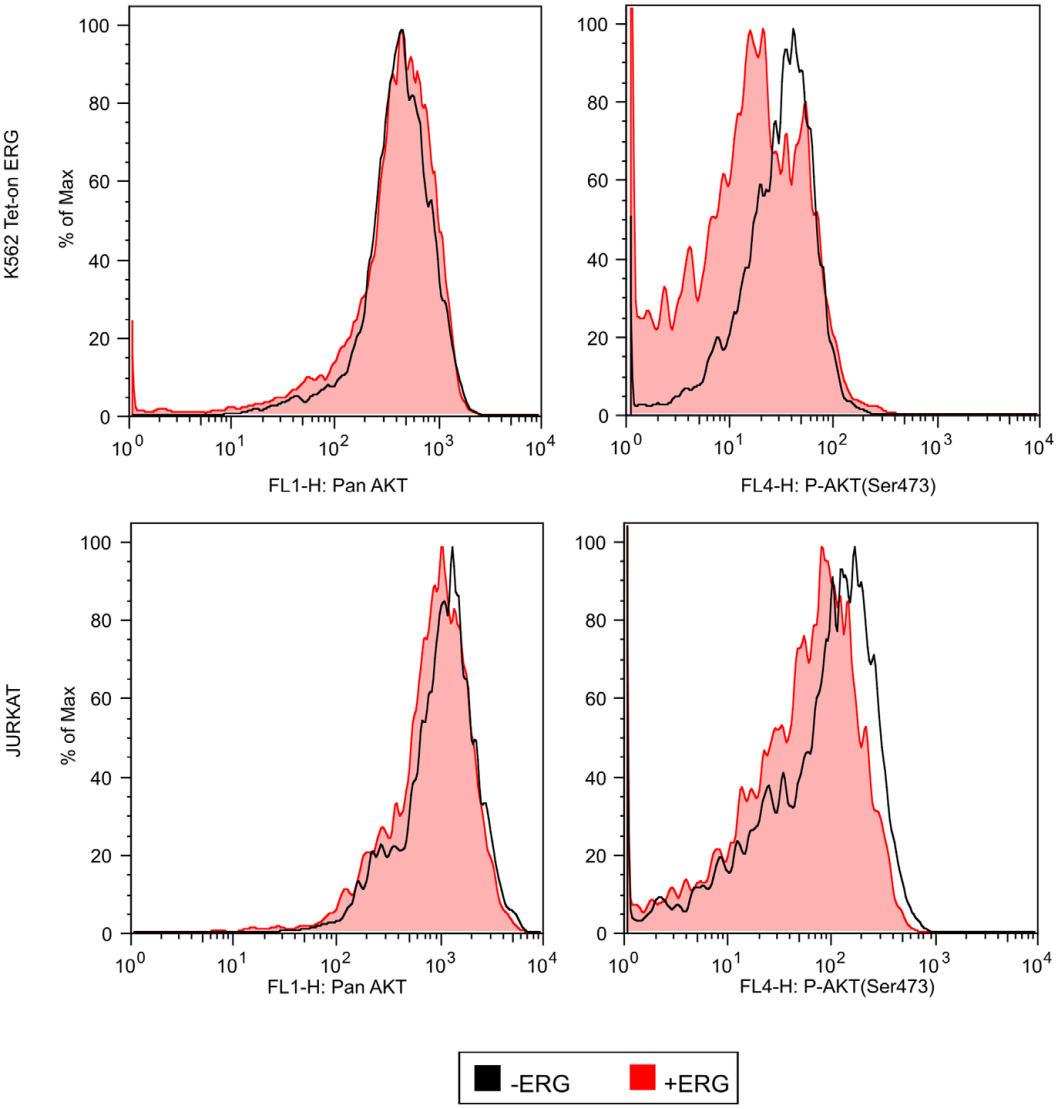
ERG induced dephosphorylation of AKT(Ser473). A histogram overlay of ERG overexpressing cells in K562 and Jurkat cells (red tinted peaks) stained for intracellular pan AKT (left side histograms) and phosphorylated AKT(Ser473, right side histograms). Non ERG expressing cells are represented by an untinted black lined peak. The histograms represent two relative AKT levels in duplicate experiments of two K562 Tet-on ERG clones. Jurkat transient transfections of pDom-empty and pDom-ERG were carried out in duplicate.

Due to the role of ERG in AKT signaling in prostate cancer [29], [30], intracellular staining for pan AKT and for active phosphorylated AKT(Ser473) was performed in leukemia. We used K562 cells transfected with the dox inducible vector system to express *ERG* [14] and measured intracellular AKT by flow cytometry. Following 5 days of dox induction, non *ERG* expressing (− Dox) cells showed greater phosphorylated AKT(Ser473) levels than *ERG* expressing (+ Dox) cells implicating that *ERG* inhibits AKT signals (Figure 4). A similar finding was observed after transient transfection of Jurkat cells using pDom-ERG and pDom-empty. A similar peak shift to the left indicated an *ERG* induced AKT(Ser473) dephosphorylation in Jurkat cells. Levels of pan AKT were unchanged in the presence or absence of ERG in both K562 Tet-on ERG clones and Jurkat cells. Due to these results, ERG may act as a repressor of AKT phosphorylation in leukemia.

### *ERG* Overexpression Induced Resistance to Multi-kinase Inhibitors

**Figure 5:**
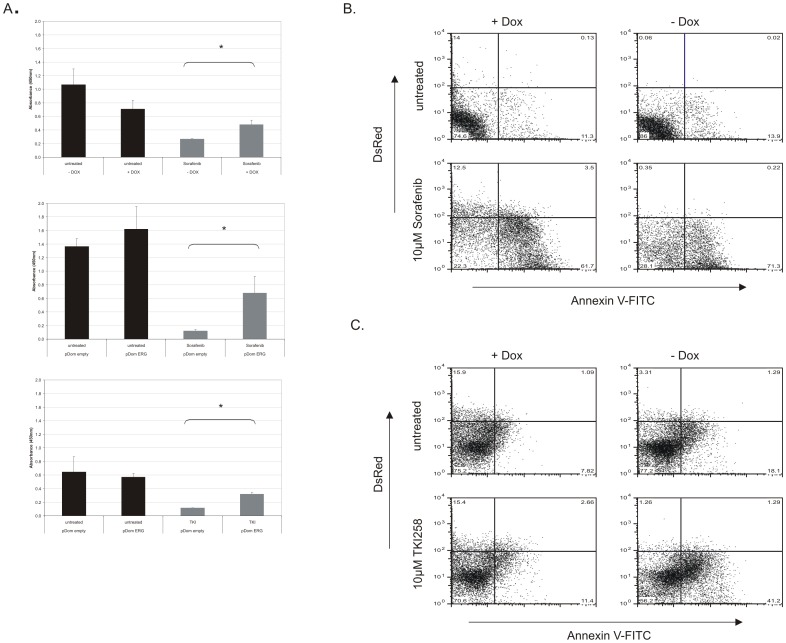
*ERG* overexpression induced resistance to multi-kinase inhibitors. A) K562 cells transduced with the inducible *ERG* expression vector (induced indicated as +DOX and uninduced indicated as –DOX) were treated with 10 µM Sorafenib in 5 parallel wells. Following 48 hours, cell proliferation was measured with WST-1 at 450 nm absorbance. Jurkat cells were transiently transfected with *ERG* expression vector, pDom-*ERG*, and the control vector pDom-empty. Twenty-four hours after seeding transiently transfected cells in 5 parallel wells, Sorafenib was added to a final concentration of 10 µM. Cell proliferation was measured 48 hours after drug addition. Cell proliferation was measured with WST-1 reagent at the 450 nm absorbance. Cell proliferation was measured with WST-1 in Jurkat cells transiently transfected with *ERG* expression vector pDom-*ERG* and the control vector pDom-empty. Twenty-four hours following transient transfection of pDom-*ERG* and pDom-empty, TKI258 was added to a final concentration of 1 µM. Cell proliferation was analyzed as described above. Bar graphs display the average of 5 experiments. Statistical significance was analyzed using Wilcoxon rang sum test. Asterisk indicates statistically significant results. *: P<0.05. **B and C)** ERG (+Dox and −Dox) K562 cells were treated with 10 µM Sorafenib or 10 µM TKI258 for 72 hours in order to determine the effects of drug induced apoptosis by Annexin V-FITC detection. This was conducted in two independent *ERG* inducible K562 Tet-on clones and one of two independent experiments is represented by dot plots.

ChIP-chip assays on primary leukemia samples revealed significantly enriched signaling pathways that execute signal transduction through kinase cascades. Kinase inhibitors as molecular directed therapies have advanced current treatment options, including Sorafenib, which is presently studied as a molecular based therapy for certain AML subtypes [31], [32]. Here, we explored the influence of *ERG* overexpression on the sensitivity to the kinase inhibitor BAY 43-9006 (Sorafenib) as multi-kinase inhibitor that targets several ligands of PI3K/AKT pathway such as: Raf-kinase, c-KIT, VEGFR1, VEGFR2, VEGFR3, FLT3 and PDGFR-β [31]. K562 Tet-on ERG cells were treated with 10 µM Sorafenib in the presence and absence of dox and proliferation was measured using the WST-1 assay. *ERG* overexpressing (+ Dox) cells treated with Sorafenib showed a near 2-fold proliferative advantage over the non *ERG* expressing (− Dox) cells treated with Sorafenib (Figure 5A).

We also validated these effects in the T-ALL cell line Jurkat using a transient transfection with an *ERG* expression vector (pDom-*ERG*) and a control vector (pDom-empty). Proliferation was measured using the WST-1 reagent at 48 hours. Similar to K562 cells, a proliferative advantage (5.5-fold) of *ERG* overexpressing Jurkat cells (pDom-ERG) treated with Sorafenib in comparison to pDom-empty Jurkat cells treated with Sorafenib was observed (Figure 5A). In addition, we investigated the sensitivity of *ERG* overexpressing cells to the receptor tyrosine kinase inhibitor, TKI258, which inhibits fibroblast growth factor receptor 3 (FGFR3), VEGFR, and PDGFR and might also inhibit other receptor tyrosine kinases such as FLT3 and c-KIT. TKI258 has already been tested in phase I and II clinical trials on advanced solid tumors and is being tested in relapsed or refractory multiple myeloma [33]. The sensitivity of *ERG* overexpressing cells (pDom-ERG vs. control pDom-empty) to TKI258 was measured (Figure 5A). A proliferative growth advantage (2.5-fold) of *ERG* overexpressing cells was observed compared to treated pDom-empty control cells, indicating ERG induced resistance to TKI258.

The ERG specific drug resistance was further verified by determining the induction of apoptosis through Annexin V-FITC staining. *ERG* overexpressing and non *ERG* expressing cells were treated with 10 µM Sorafenib for 48 hours. Untreated ERG overxpressing cells (+ Dox) and non ERG expressing cells (− Dox) had a baseline of 11.4% and 13.9% Annexin V levels. With Sorafenib treatment, *ERG* overexpressing population (DsRed positive and Annexin V negative) were sustained despite Sorafenib addition (12.5% treated compared to 14% untreated DsRed cells), with only a marginally apoptotic DsRed population of 3.5%. In contrast, the DsRed negative population of both +Dox and –Dox treated groups had higher levels of apoptotic induction with Sorafenib treatment of 61.7% and 71,5%, respectively (Figure 5B).

Treatment with TKI258 (10 µM) also resulted in the maintenance of DsRed single positive population at 15% (untreated and treated) with only a marginal portion of cells measuring Annexin V positive (DsRed+/Annexin+2.6%). In contrast, non *ERG* expressing cells (−Dox) treated with TKI258 resulted in greater induction of apoptosis (42.5%; Figure 5C). These results indicate that overexpression of *ERG* results in resistance of apoptosis to two types of kinase inhibitors multi-kinase inhibitors, Sorafenib and TKI258. This finding of ERG dependent drug resistance moreover supports our conclusion from the ChIP-chip of primary leukemia specimens that ERG contributes to deregulating kinase signaling.

## Discussion

The ETS family of transcription factors regulates functions essential for hematopoietic development [34]. Aberrant expression of these genes has been observed with hematologic malignancies and solid tumors [35]. Additionally, high *ERG* mRNA expression at diagnosis is an independent negative prognostic factor in cytogenetically normal AML and T-ALL [7], [10]. The role of ERG in leukemia is also demonstrated by the proliferative potential and ability of ERG to promote myeloid and lymphoid leukemias. Hence, it is of great interest to identify genes, functions and pathways differentially regulated by ERG in leukemogenesis.

Herein, we conducted ChIP-chip analyses in primary AML samples, T-ALL and nBM sample. Functions enriched in nBM were cell cycle and tissue development, suggesting these to be physiological functions in hematopoietic cells regulated by ERG. The most distinguishing functions enriched in primary leukemia cells were: growth and proliferation, cellular development, cell morphology, and cellular movement, all of which have been associated with ERG’s molecular function. ChIP-chip analysis of ERG bound targets in the T-ALL cell line Jurkat revealed development, adhesion, and stem cell pluripotency [14], which in this study was also confirmed in primary leukemia. Furthermore, we uncovered *DAAM1*, a WNT signaling and potent morphogenesis gene, and *NUMB* as novel putative targets of ERG in leukemia. *DAAM1* function is mapped in planar cell polarity pathway with an impact on cell migration however a role in AML biology has not been described. For *NUMB*, a potent inhibitor of NOTCH1, its expression was shown to be significantly down regulated in acute myeloid leukemia [36]. In broader terms, it has also been reported that low *NUMB* mRNA expression array of various cell lines from tumor types treated with AKT/PI3K inhibitors correlates with resistance to PI3K/mTOR inhibition [25]. Thus we hypothesize that *ERG* overexpression may repress *NUMB* possibly through negative regulation of AKT/PI3K in acute leukemia.

We biologically categorized ChIP-chip enriched gene datasets for signaling pathways. Interestingly, ChIP-chip experiments for ERG target genes in primary leukemia revealed specifically enrichment of the PI3K/AKT pathway. The PI3K/AKT pathway regulates cell proliferation and is frequently activated in human cancer [37]. A recent study also demonstrated that co-activation of the PI3K/AKT pathway and *ERG* overexpression collaborated with prostate specific androgen response (AR) and lack of PTEN in the development of prostate carcinoma [30]. In our studies, prostate cancer specific genes such as *TMPRSS2* and *AR* have been enriched in leukemia ChIPs, although transcriptional regulation by *ERG* was not detectable at the mRNA level (data not shown). Constitutive activation of the PI3K/AKT pathway is a frequent event in AML due to mutations of upstream targets such as *FLT3*, c-*KIT* or *RAS* [38]. In comparison to prostate studies where ERG overexpression did not induce phosphorylation of AKT [30], we found that *ERG* overexpression resulted in dephosphorylation AKT(Ser473) suggesting that *ERG* overexpression represses or bypasses AKT activation. Thus, ERG overexpression without constitutive AKT activation leads one to believe that alternate parallel signaling pathways are responsible for ERG mediated kinase resistance in leukemia.

Studies have shown that Sorafenib inhibits FLT3, c-KIT or RAF, which are upstream of PI3K/AKT. TKI258 also possibly targets FLT3 and c-KIT. Hence, we explored ERG dependent proliferation effects of Sorafenib, a multi-kinase inhibitor, and TKI258, a receptor tyrosine kinase inhibitor, on *ERG* overexpressing cells. We could show a proliferative advantage of *ERG* overexpressing cells in comparison with non *ERG* expressing cells when treated with Sorafenib or TKI258. *ERG* expressing cells traceable by DsRed were maintained demonstrating resistance to apoptosis. This ERG mediated resistance to tyrosine kinase inhibitors might also contribute to shunting PI3K/AKT signals to other pathways although these intrinsic resistances have not yet been defined. More importantly, this report implicates that PI3K/AKT inhibitors may not be useful for patients with high *ERG* expression in acute leukemia.

Interestingly, another significantly enriched biological theme was the p53 signaling pathway. Thus far, little is known with regards to ERG and p53 signaling in acute leukemia, although ERG does affect p53 acetylation status in prostate cancer studies [39]. Further evaluation of a possible ERG-mediated p53 signaling pathway regulation will also enhance the understanding of ERG-related role in leukemia.

In summary, ChIP-chip, bioinformatic analysis, and functional assays reveal that ERG may modulate kinase signaling pathways. Additionally, we demonstrate that ERG overexpressing cells are resistant to the multi-kinase inhibitor Sorafenib and tyrosine kinase inhibitor, Dovitinib to support the ERG specific drug resistance. Furthermore, we propose that ERG driven drug resistance override PI3K/AKT signaling by alternate pathways. These alternate pathways need to be further investigated for effective drug design and adapted therapies for ERG overexpressing high-risk leukemias.

## Supporting Information

Figure S1 Motif analysis with the Pscan software and enriched biological functions. **A)** Demonstrated is the significant enrichment of the ETS motif conducted for the 7 ChIP-chip experiments. Displayed is a presentative graphical analysis of GGAA/T motif for AML D. The height of the bases in the motif corresponds to significance in relation to the neighboring bases. **B)** Displayed are the biological functions that were enriched in at least three of seven primary leukemia samples by ChIP-chip. The bars represent the negative logarithmic function of the Benjamini-Hochberg P-value.
(TIF)

Table S1 Description of bone marrow specimens used in this study. The table describes relevant characteristics of bone marrow donor specimens used in this study such as donor sex, age, ERG mRNA expression, FAB classification, cytogenetic profile, and molecular genetic characteristics.
(TIF)
