# Supplementary figures and images for: ERG Transcriptional Networks in Primary Acute Leukemia Cells Implicate a Role for ERG in Deregulated Kinase Signaling
Source: PLoS One. 2013 Jan 3;8(1):e52872. doi: 10.1371/journal.pone.0052872 (PMC3536782; doi:10.1371/journal.pone.0052872)

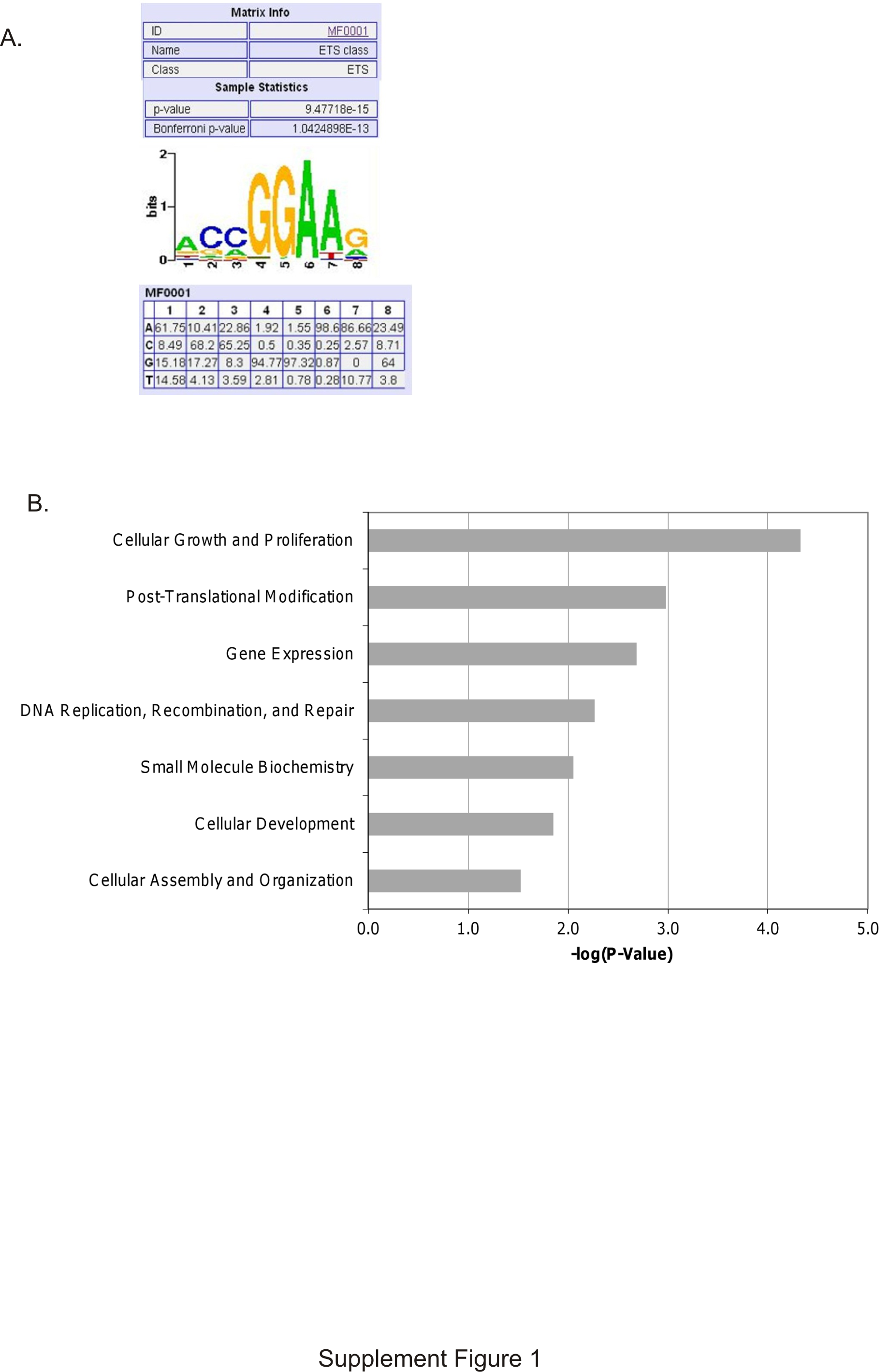

Supplement: Figure S1 — Motif analysis with the Pscan software and enriched biological functions. A) Demonstrated is the significant enrichment of the ETS motif conducted for the 7 ChIP-chip experiments. Displayed is a presentative graphical analysis of GGAA/T motif for AML D. The height of the bases in the motif corresponds to significance in relation to the neighboring bases. B) Displayed are the biological functions that were enriched in at least three of seven primary leukemia samples by ChIP-chip. The bars represent the negative logarithmic function of the Benjamini-Hochberg P-value. (TIF) [file pone.0052872.s001.tif]

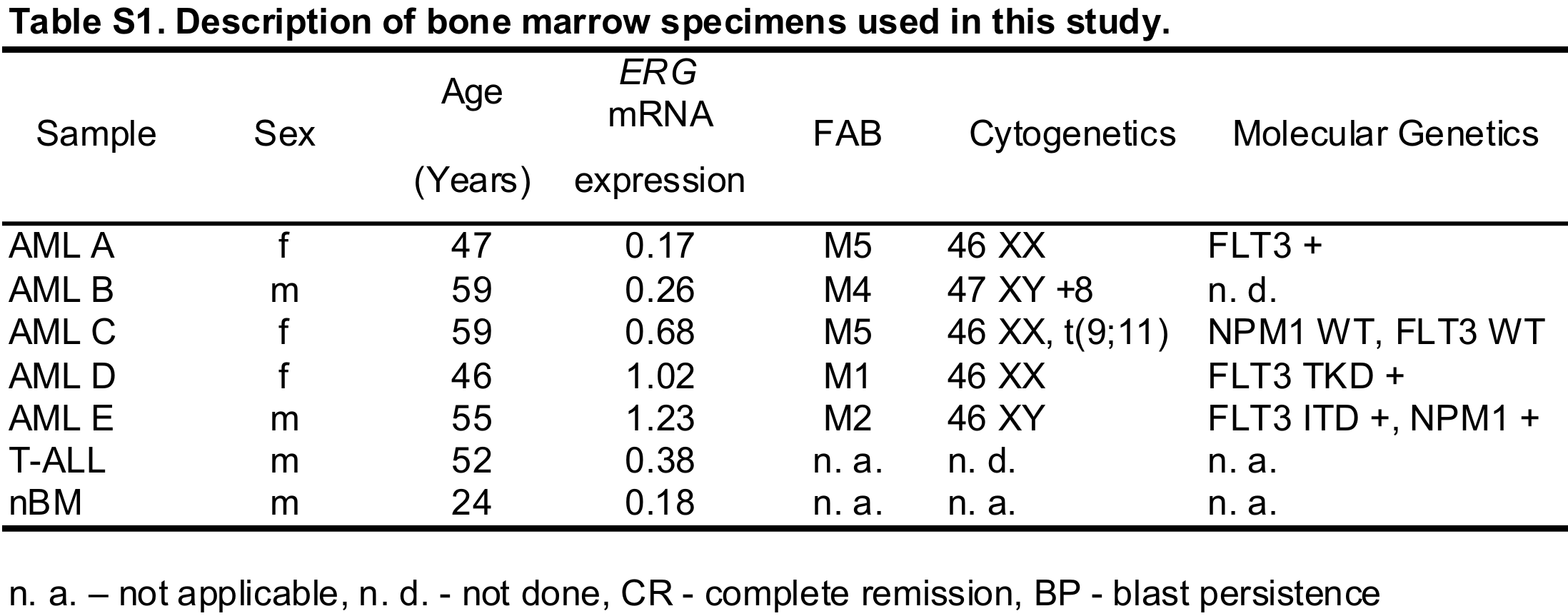

Supplement: Table S1 — Description of bone marrow specimens used in this study. The table describes relevant characteristics of bone marrow donor specimens used in this study such as donor sex, age, ERG mRNA expression, FAB classification, cytogenetic profile, and molecular genetic characteristics. (TIF) [file pone.0052872.s002.tif]
